# Running to get “lost”? Two types of escapism in recreational running and their relations to exercise dependence and subjective well-being

**DOI:** 10.3389/fpsyg.2022.1035196

**Published:** 2023-01-25

**Authors:** Frode Stenseng, Ingvild Bredvei Steinsholt, Beate Wold Hygen, Pål Kraft

**Affiliations:** ^1^Department of Education and Lifelong Learning, Norwegian University of Science and Technology, Trondheim, Norway; ^2^Oslo University College, Oslo, Norway; ^3^Department of Psychology, University of Oslo, Oslo, Norway; ^4^Norwegian University of Science and Technology Social Research, Trondheim, Norway

**Keywords:** addiction, flow, coping, emotion regulation, affect

## Abstract

Escapism is a fundamental motivation in many forms of activity engagements. At its core, *escapism* is “a habitual diversion of the mind … as an escape from reality or routine”. Accordingly, escapism may entail many adaptive and maladaptive psychological antecedents, covariates, and outcomes. However, few studies have been conducted on escapism as a motivational mindset in running. Here, in a sample of recreational runners (*N* = 227), we applied a two-dimensional model of escapism, comprising *self-expansion* (adaptive escapism) and *self-suppression* (maladaptive escapism), and examined how they were related to exercise dependence and subjective well-being. First, confirmatory factor analyses showed that the escapism dimensions were highly diversifiable in the sample. Then, correlational analyses showed that self-expansion was positively correlated to subjective well-being, whereas self-suppression was negatively related to well-being. Self-suppression was more strongly related to exercise dependence compared to self-expansion. Finally, path analyses evidenced an explanatory role of self-expansion and self-suppression in the inverse relationship between exercise dependence and well-being. In conclusion, the present findings support escapism as a relevant framework for understanding the relationship between exercise dependence in running and subjective well-being.

## Introduction

Three decades ago, Baumeister (1991) emphasized the motivational powers of escapism, but the term has mostly been a *rara avis* in the landscape of motivational sciences. In fact, it does not seem to have been empirically explored in relation to exercise or sport engagement at all, only in the consumption of sport as entertainment (Astakhova et al., 2022). In contrast, the interest in escapism as a distinct form of motivation has escalated in other areas. For instance, escapism has been used as a framework for idiosyncratic experiences in cultural sciences, such as cinematography (Addis and Holbrook, 2010), science of art (Walmsley, 2011), and media science (Wulf et al., 2022). Most importantly, escapism has been incorporated into the clinical assessment of pathological gaming (American Psychiatric Association, 2013; Petry and O'Brien, 2013). One criterion for the assessment of Internet gaming disorder is “use of Internet games to escape or relieve a negative mood (e.g., feelings of helplessness, guilt, and anxiety)” (American Psychiatric Association, 2013, p. 795). The inclusion of this escapism criterion was greatly debated, as escapism does not necessarily coincide with a pathological interest or with detrimental outcomes (Aarseth et al., 2017; Kuss et al., 2017; Hussain et al., 2021).

At its very core, *escapism* is a “habitual diversion of the mind to purely imaginative activity or entertainment as an escape from reality or routine” (Merriam-Webster, n.d.), or “an activity, a form of entertainment, etc. that helps you avoid or forget unpleasant or boring things” (Oxford University Press, 2011). Undoubtedly, these definitions do not infer exclusively maladaptive engagement causing negative consequences, supporting the criticism related to applying escapism as a unidimensional construct (Kardefelt-Winther, 2015).

As in the case of gaming, some people develop a pathological interest in running. About one in four recreational runners (Egorov and Szabo, 2013) and 40% of competitive runners (Juwono et al., 2021) show signs of exercise addiction. What remains unclear, however, is how psychological dependence in running relates to escapism’s darker and brighter sides and how these constructs relate to well-being. Thus, in the present study, by applying a two-dimensional operationalization of escapism (Baumeister, 1990, 1991; Stenseng et al., 2012, 2021) in a sample of recreational runners, tapping into both the darker and the brighter sides of escapism, we investigated to which extent the two facets of escapism were differently related to exercise dependence and subjective well-being.

### The two-dimensional model of escapism and its relevance for physical exercise

Baumeister (1990, 1991) explored escapism in light of *selfhood*, that is, people’s innate ability to monitor, assess, and reflect upon their self-presentations, aims and goals, and their own identity. With this ability for selfhood, it follows that our failures and losses also may be scrutinized in hindsight, which may lead to considerable distress, and when becoming disturbing to an intolerable extent, even lead people into suicidal behavior (Baumeister, 1990; Vohs and Baumeister, 2002). Selfhood may also lead to considerable distress for future occurrences, due to humans’ ability to imagine prospective situations and outcomes (Blouin-Hudon and Pychyl, 2015). According to Baumeister (1991), humans seek ways to unwind from this constant self-consciousness through endorsement of activities that pull focus away from their self, if just for their own psychological reward. Moreover, such escapes from self are embedded in our popular culture, such as supporting one’s favorite soccer, hockey, or basketball team every Sunday, going to the movies or concerts, or binging Netflix series (Stenseng et al., 2021). However, no efforts to operationalize these forms of everyday escapism were suggested by Baumeister (1991) and his colleagues based on their theoretical work, which stalled empirical investigations of these ideas.

One way of losing oneself is to engage in physical activity, which normally facilitates continuous focus on specific tasks, rules, and actions. The state of flow (Csikszentmihalyi, 1990) has often been used to describe the psychological condition of being cognitively and emotionally absorbed into activities, which has been highlighted as a motivational factor in sport activities. The *flow* state is defined as an engagement in which one’s abilities transcend the demands of the activity, which leads to intense cognitive focus, alertness, and a feeling of mastery and joy (Csikszentmihalyi and LeFevre, 1989; Csikszentmihalyi, 1990). A self-report questionnaire was developed to assess nine aspects of flow (see Jackson and Marsh, 1996), which facilitated psychometrical assessments of flow that have spurred a large body of empirical studies (for a review, see Stamatelopoulou et al., 2018). In many respects, flow resembles the state of escape (Stenseng et al., 2012), and correspondingly, it has also been argued that flow has a darker side, instigating an addiction-like interest (Partington et al., 2009). Nevertheless, the concept of flow does not take into account the psychological drive behind the flow-producing activity engagement, and as such, does not fully illuminate the “escape” motives embedded in escapism, a prerequisite clearly stated in the definitions of the term.

Stenseng et al. (2012) presented a two-dimensional model of escapism and developed a corresponding self-report scale (the Escapism scale, see Stenseng et al., 2012, 2021). In their model, the dimensions Self-Suppression and Self-Expansion were identified, stemming from antagonistically different motivational mindsets. Self-Suppression is rooted in *prevention motives* (Higgins, 1998), in the sense that the individual engages in an activity to prevent, or suppress, troublesome thoughts or emotions. With this type of escapism motivation, the main goal of the activity engagement is to distract oneself from uncomfortable mental processes, such as thinking about future challenges and/or rumination about the past (“repetitive, prolonged, and recurrent negative thinking about one’s *self*, feelings, personal concerns and upsetting experiences;” Watkins, 2008, p. 164). When these mental processes stir negative emotions, individuals become triggered to suppress and/or alleviate them, and a common response is to dim these troublesome thoughts by pursuing experiences that may outshine them, at least momentarily. A similar motivation is seen in procrastination, simply defined as “putting off for tomorrow what one should have done today” (Milgram et al., 1988, p. 197), where individuals avoid an urgent task due to lack of a proactive mindset; simultaneously, the task becomes more and more pressing, and the situation more uncomfortable. However, the suppression of negative emotions also dampens positive ones (Gross, 2002), resulting in forms of engagement, independent of type of activity, that are less likely to be accompanied by positive effect. As shown by Stenseng et al. (2012, 2021), self-suppression is related to more trait emotion suppression and lower self-control, as well as fewer positive emotions in activity engagement. Importantly, self-suppression is also negatively related to subjective well-being (Stenseng et al., 2012, Studies 1 and 2; Stenseng et al., 2021).

With the other type of escapism, self-expansion, the motivation is rooted in *promotion motives* (Higgins, 1998). Individuals who engage out of motives to promote positive emotions are likely to gain more positive effect during the activity engagement, but they also experience more long-term benefits from the engagement. This type of motivation is related to more harmonious passion (Stenseng and Phelps, 2016); that is, an interest in an activity that nourishes one’s subjective well-being (Vallerand et al., 2003). It has also been shown to be associated with more flow experiences in the activity, compared to self-suppression (Stenseng et al., 2012, Study 3; Stenseng and Phelps, 2016). Moreover, self-expansion has been shown to be related to more basic need satisfaction (e.g., autonomy, relatedness, competence; see Deci and Ryan, 2000) during gaming (Stenseng et al., 2021, Study 1), as well as more positive affect experienced during the activity and higher general subjective well-being (Stenseng et al., 2012, Study 1). Also, when related to online streaming, self-expansion was related to an approach-oriented coping style (Moritz et al., 2016), which normally means acknowledging the challenges and then actively seeking to resolve them, whereas self-suppression was more strongly related to avoidance coping (Stenseng et al., 2021, Study 2), which is characterized by procrastination, suppression, or other measures to temporarily avoid the problem cognitively, in concordance with the *cognitive appraisal hypothesis* of psychological dependence (Szabo, 1995; see also Berczik et al., 2012).

In sum, there is considerable support for a two-dimensional operationalization of escapism, one maladaptive and one adaptive, across several types of activities. The two dimensions, Self-Suppression and Self-Expansion, are differently related to, for example, trait self-regulation, flow and effect in the activity, and subjective well-being. Still, no empirical studies have explored this model in relation to running, which truly is an activity where different psychological mindsets and states are evident (Masters and Lambert, 1989; Siebers et al., 2022), and which also is an activity with enormous popularity (Pereira et al., 2021).

### Escapism in relation to exercise dependence and life satisfaction

As argued above, escapism may either be nourishing for one’s subjective well-being, or a threat to it, depending on which motivational mindset the escapism behavior reflects. In its maladaptive form, escapism appears to overlap with psychological characteristics of exercise addiction or exercise dependence (for a differentiation of these two, see Szabo et al., 2018).

First, when it comes to personality variables, both self-suppression and exercise dependence are related to lower self-control (Stenseng et al., 2012, Study 1; Zimanyi et al., 2021), but higher trait emotion suppression (Stenseng et al., 2012; Kun et al., 2022, Study 1), and avoidance coping (Egorov and Szabo, 2013; Stenseng et al., 2021, Study 2). Second, both are related to negative effect, or lack of positive effect, during activity engagement as well as after (Hausenblas and Symons Downs, 2002; Stenseng et al., 2012, Study 1; Stenseng et al., 2021, Study 1), as opposed to self-expansion. Third, both self-suppression and exercise dependence are related to intra-and interpersonal conflicts (Stenseng et al., 2012, Study 1; Stenseng et al., 2015). Fourth, both are related to obsessive passion for the activity, whereas self-expansion appears unrelated (Paradis et al., 2013; Stenseng and Phelps, 2016). Finally, both concepts are related to diminished life satisfaction (Hausenblas and Symons Downs, 2002; Stenseng et al., 2012, 2021).

The above-mentioned studies highlight the empirical overlap between self-suppression escapism and exercise dependence. However, these two concepts are anchored in quite different theoretical underpinnings. Most research on exercise dependence is focusing on the phenomenon itself; that is, addiction-like engagement in the activity and its correlational ties to personality characteristics (e.g., self-control, perfectionism, and coping) and as a framework to assess prevalence of this type of addiction across different populations (e.g., Lichtenstein et al., 2014). These empirical investigations have established exercise dependence as a sound, valid phenomenon, with both general and clinical relevance (Hausenblas and Symons Downs, 2002; Terry et al., 2004). However, by predominantly focusing on addiction-like sides of exercise dependence, inspired by other addictions, such as alcoholism (Szabo et al., 2015), this research does not fully illuminate why people maintain their maladaptive activity engagement, although some theoretical alleyways have been suggested (see Berczik et al., 2012; Egorov and Szabo, 2013). This is, after all, the great paradox: why do people keep on engaging in something that is detrimental to their emotional well-being and to their social bonds? What are the psychological nutrients in intense activity engagement that are so rewarding that people keep on doing what is bad for them?

The “state of escape” is arguably one such nutrient in activity engagements (Baumeister, 1991; Stenseng et al., 2012), and it may thus partly explain the paradoxical behavior of exercise dependence. However, in contrast to exercise dependence, the two-dimensional model of escapism targets two separate routes toward the escape experience, and thus suggests that the mere psychological experience in the activity must be seen in light of its motivational mindsets (prevention vs. promotion). To our knowledge, no studies have explicitly studied running from such a dualistic conceptualization of escapism. In fact, very few researchers have explored escapism in running at all. As an exception, Kerrigan et al. (2014) conducted interviews with runners on how musicalization affected their running experiences. These researchers found several examples on how participants used music to create soundscapes to their running, which promoted their focus during running and thus enhanced their experience of pleasurable escapism in running. Besides this one study, escapism as a motivational factor in running seems to remain unexplored.

## The present study

Introductorily, we have described the two-dimensional model of escapism (Baumeister, 1991; Stenseng et al., 2012, 2021) and justified the model’s potential relevance for exercise dependence and subjective well-being. Moreover, we pointed at the lack of studies on escapism in running. Accordingly, in this cross-sectional study, in a sample of recreational runners, we first explored the two-dimensional operationalization of escapism as suggested by Stenseng et al. (2012, 2021) using their 14-item Escapism scale. Second, we investigated correlational overlaps between the two escapism dimensions, Self-Suppression and Self-Expansion, to determine their differential relations to exercise addiction and subjective well-being, as well as gender, age, and weekly amount of running. Third, we conducted moderation analyses by means of path modeling to test the additive explanatory effect of the escapism dimensions in explaining the inverse relationship between exercise dependence and well-being, including gender, age, and weekly amount of running as control variables.

## Materials and methods

### Procedure and participants

Participants were recruited from social media sites for individuals with a particular interest in running (e.g., www.facebook.com/groups/lopeprat). Administrators for these sites were contacted by email to ask for permission to include the link to our online survey questionnaire in their news feed or in their forum. In addition, to recruit additional participants, a sponsored Facebook post was published with the inclusion question: Are you a regular runner? If the response was “yes,” they were forwarded to our questionnaire. Ethical concerns were addressed on the first page of the questionnaire, including confirmation of informed consent. On beforehand, the authors aligned the project with the internal policy for research ethics at the respective university. Also, a clinical psychologist was accessible to the participants throughout data collection per e-mail. Only fully answered questionnaires were submittable, ensuring data without missing values. The final sample consisted of 227 regular runners, 115 women and 112 men, with a mean age of 42.7 years (*SD* = 11.1 years). On average, they spent approximately 5 h on running per week (*SD* = 2.54). Nine participants ran more than 10 h per week.

### Measures

#### Dimensions of escapism

The Escapism scale (Stenseng et al., 2012) was used to measure two sets of escapism motives: Self-Suppression and Self-Expansion. The scale has previously been applied to several types of activities, such as sports, gaming, and entertainment (Netflix, HBO, etc.; Stenseng et al., 2015, 2021), and it has demonstrated good factorial validity and internal consistency in all samples. The full scale consists of 14 items, five items to measure Self-Expansion, six items to measure Self-Suppression, and three escapism criterion items. The criterion items tap into the state of escape, which characterizes both dimensions of escapism, as well as time narrowing, cognitive focus, and immersion, and they should thus correlate with both subscales. All items are phrased as an extension of the stem “When I engage in the activity…,” which in this case was modified to “When I am running…” to correspond with the activity in focus in the present research. Sample items for Self-Expansion are “I try to learn new things about myself,” and “I open up for experiences that enrich my life.” Sample items for Self-Suppression are “I try to forget the difficult things in my life,” and “I try to suppress my problems.” Responses were made on a 5-point Likert scale ranging from *totally disagree* to *totally agree*. Cronbach’s alphas were 0.78 for Self-Expansion and 0.86 for Self-Suppression. A composite score of the criterion items correlated 0.54 with Self-Expansion and 0.27 with Self-Suppression, both significant on the 0.01 level.

#### Exercise dependence

The Exercise Dependence Scale (Hausenblas and Symons Downs, 2002) was used to measure addiction to running. The Exercise Dependence Scale is adapted from the *DSM-IV* (American Psychiatric Association, 1994) assessment for substance dependence, based on seven criteria: (a) tolerance, (b) withdrawal, (c) intention effect, (d) lack of control, (e) time, (f) reduction in other activities, and (g) continuance. Sample items are “I am unable to reduce how long I exercise” and “I would rather exercise than spend time with family/friends.” Responses were made on a 6-point Likert scale, ranging from *never* to *always*. Cronbach’s alpha was 0.89.

#### Subjective well-being

The Satisfaction With Life Scale (Diener et al., 1985) was used to measure subjective well-being among participants. This is a five-item scale addressing cognitive evaluations of current life satisfaction, including items such as “In most ways my life is close to my ideal” and “I am satisfied with my life.” Responses were made on a 7-point Likert scale, ranging from *totally disagree* to *totally agree*. Cronbach’s alpha was 0.9.

## Results

### The factorial structure of the escapism scale

Although the Escapism scale (Stenseng et al., 2012) has proven relevant across several types of activities, a factorial assessment of the scale was performed in order to examine its performance in relation to running specifically. Thus, a confirmatory factor analysis was performed in Mplus 8.01 (Muthén, 2016). The two-factor solution was examined by means of maximum likelihood estimation, which resulted in good model fits: *χ*^2^(43) = 74.78, *p* = 0.002, RMSEA = 0.057, SRMR = 0.052, CFI = 0.997, TLI = 0.992. Standardized factor loadings for items on the Self-Expansion dimension ranged from 0.84 to 58 and from 0.83 to 0.55 on the Self-Suppression dimension (see Table 1). Results supported the two-dimensional operationalization of escapism embedded in the Escapism scale and that this motivational dualism is found in recreational running.

**Table 1:** Factor loadings for the escapism dimensions, self-expansion and self-suppression, from confirmatory factor analysis performed in Mplus 8.01.

When I run…	Self-expansion	Self-suppression
1. I try to learn new things about myself.	**0.84**	0.07
2. I often surprise myself in a positive way.	**0.64**	0.03
3. I open up for experiences that enrich my life.	**0.60**	0.17
4. I am filled with positive energy that transfers to other parts of my life.	**0.60**	0.08
5. I try to get to know myself better.	**0.58**	0.05
6. I try to forget the difficult things in my life.	0.06	**0.83**
7. I try to suppress my problems.	0.10	**0.79**
8. I want to escape from reality.	0.05	**0.78**
9. I want to escape from myself.	0.01	**0.76**
10. I shut out the difficult things I do not want to think about.	0.15	**0.61**
11. I try to prevent negative thoughts about myself.	0.19	**0.55**

Bold value is standard procedure of only visual character to emphasize the factor structure.

### Bivariate correlations

Bivariate correlation analyses showed, most notably (see Table 2), that Self-Expansion was positively correlated with subjective well-being (*r* = 0.17, *p* < 0.05), whereas Self-Suppression was negatively correlated (*r* = −0.38, *p* < 0.01). Somewhat surprisingly, both Self-Expansion (*r* = 0.34, *p* < 0.01) and Self-Suppression (*r* = 0.53, *p* < 0.01) were positively correlated with exercise dependence, but to substantially different degrees. Self-Expansion and Self-Suppression were only moderately correlated (*r* = 0.22, *p* < 0.01). Of notice, Self-Expansion and Self-Suppression were not differently related to gender (*r* ≠ *p* = 0.46), age (*r* ≠ *p* = 0.37), or amount of running (*r* ≠ *p* = 0.08).

**Table 2:** Means, standard deviations, and bivariate correlations from Pearson’s *r*-analyses performed in SPSS 25.

	*M*	*SD*	1	2	3	4	5	6	7
1. Gender	0.51	0.50	-						
2. Age	42.66	11.12	−0.14^*^	-					
3. Hours spent running	4.84	2.54	−0.14^*^	−0.09	-				
4. Self-expansion	3.78	0.65	0.11	−0.21^**^	0.15^*^	-			
5. Self-suppression	2.23	0.55	0.10	−0.24^**^	0.02	0.22^**^	-		
6. Exercise dependence	2.29	0.55	0.05	−0.26^**^	0.47^**^	0.34^**^	0.51^**^	-	
7. Subjective well-being	5.21	1.24	0.09	0.07	−0.04	0.17^*^	−0.38^**^	−0.30^**^	-

*p < 0.05, **p < 0.01

In sum, the correlational analyses supported the two-dimensional conceptualization of escapism, in the sense that Self-Expansion and Self-Suppression were substantially differently correlated to exercise dependence and subjective well-being, but not differently related to age and gender, and they only modestly overlapped each other.

### Path analyses

As suggested in the introduction, the two sets of escapism motives could play an explanatory role in how exercise dependence and life satisfaction are empirically entangled. To test this more dynamic approach to the data, which calls for the control of overlapping variance between study variables, we turned to path analyses (Kline, 2015). We are aware of the distinct limitations restricting any causal inferences from path analyses on cross-sectional data (Kline, 2015), and we emphasize that this approach was taken to give a clearer image of the interrelations of the variables, not their true causal structure.

**Figure 1:**
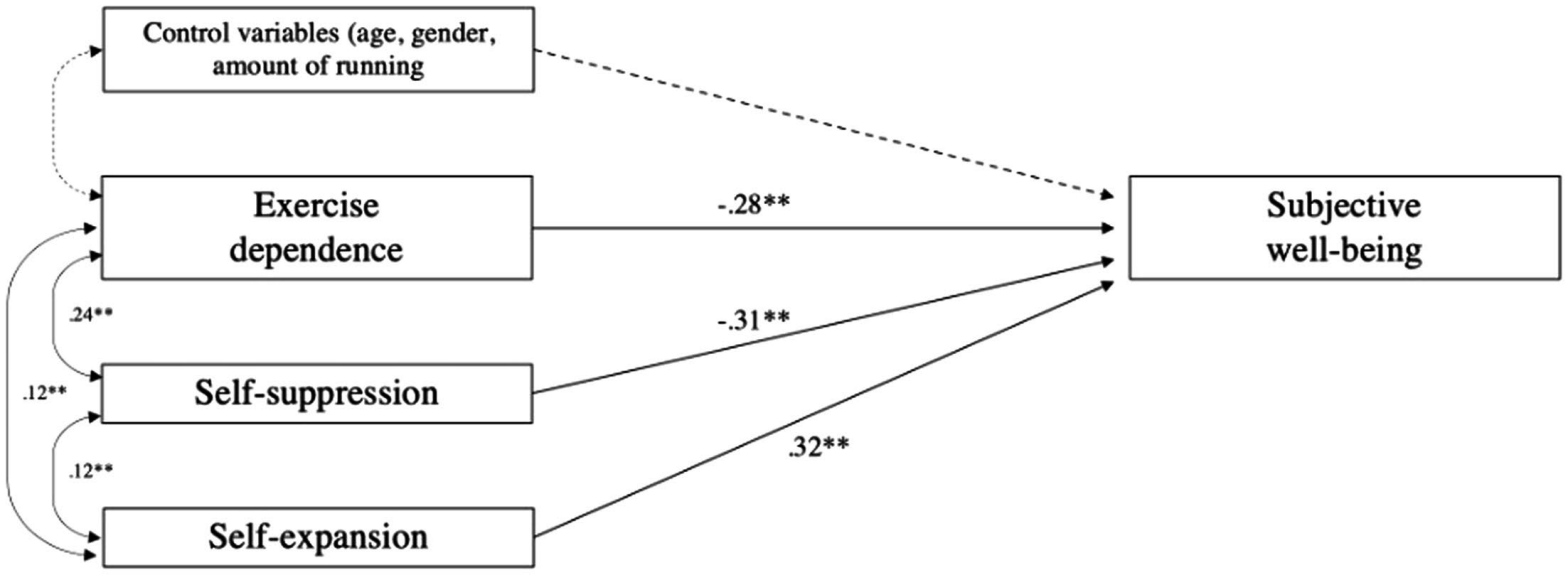
Path coefficients (Standardized Betas) and covariates for the full path model tested in AMOS 28. **p < 0.01.

A structural path model was defined in AMOS 28 from the theoretical assumptions outlined in the introduction: Exercise dependence hampers subjective well-being, but the two escapism dimensions add explanatory power to this relationship, controlling for gender, age, and amount of running per week. First, a simple model was tested, containing only exercise dependence as predictor and subjective well-being as outcome, but also including each of the control variables. As expected, a significant relationship was found, also when controlling for these third variables (*ß* = −38., *p* < 0.001). Then, when including both Self-Expansion and Self-Suppression as predictors in the model, with additional covariates between all endogenous variables, the relationships between exercise dependence and well-being waned but were still significant (*ß* = −0.28, *p* < 0.001). However, both Self-Suppression (*ß* = −0.31, *p* < 0.001) and Self-Expansion (*ß* = 0.32, *p* < 0.001) had significant predictive value in the model, and in opposite directions (negative vs. positive, respectively), emphasizing their explanatory role in predicting the variance in well-being over and above exercise dependence (see Figure 1).

## Discussion

The aims of the present study were threefold:

1. Test a two-dimensional model of escapism in relation to recreational running.
2. Investigate the relations of the two escapism dimensions, Self-Suppression and Self-Expansion, to exercise and subjective well-being and determine their common or differentiated links to gender, age, and amount of running.
3. Test the additive role of the two escapism dimensions in the inverse relationship between exercise dependence and subjective well-being.

First, the factor analyses confirmed a two-dimensional structure of escapism in running. The analyses conveyed an overall structure with good model fits, two distinctive subdimensions, and a substantial proportion of explained variance. Dimensions also correlated similarly with the escapism criterion items and displayed high internal reliability. Results were in accordance with previous findings in other activities, such as leisure activities in general, gaming, swimming, cycling, and streaming (Stenseng et al., 2012, 2021; Stenseng and Phelps, 2016), thus supporting the applicability of the Escapism scale across a variety of activities.

Second, the correlational analyses showed that the two escapism dimensions, Self-Suppression and Self-Expansion, overlapped differently with exercise dependence and life satisfaction. However, these two dimensions were not differently related to age, gender, or amount of time spent running per week. Findings supported the adaptive versus maladaptive dichotomy of Self-Expansion and Self-Suppression, in that Self-Suppression was more strongly related to exercise dependence and lower well-being than Self-Expansion. Importantly, these results show that escapism mindsets are rooted in latent psychological processes more than descriptive characteristics of individuals, such as age, gender, and time spent running.

Third, as tested through path analyses, both Self-Suppression and Self-Expansion moderated the inverse relationship between exercise dependence and subjective well-being, demonstrating their role beyond exercise dependence in explaining individual differences in well-being. As such, these findings support the idea that these escapism mindsets are relevant in explaining both addiction-like engagement in running, as well as levels of general well-being.

As mentioned, escapism as a motivational factor in activity engagements has been highlighted by several authors (Baumeister, 1991; Wulf et al., 2022) and has recently been extensively debated in relation to the inclusion of Internet gaming disorder in *DSM-5* and *ICD-11* (Aarseth et al., 2017). Nevertheless, empirical investigations of this phenomenon in gaming have been rather rare, and somewhat led to ambiguous findings (Hussain et al., 2021). One explanation for this may be that escapism almost exclusively has been operationalized as a unidimensional phenomenon (for an exception, see Hagström and Kaldö, 2014). Although Baumeister (1991) and others (e.g., Hussain et al., 2021) have argued that escapism may have both adaptive and maladaptive underpinnings and outcomes, a two-dimensional operationalization of escapism across activities has been lacking. The present validation of the Escapism scale in a sample of runners, however, adds empirical support to the applicability of a two-dimensional representation of escapism, which to a greater extent seems to capture the totality of escapism as a phenomenon.

The present findings are relatable to exercise dependence in many respects. Exercise dependence, and in this case in relation to running, shares several similarities with other types of addictions. It shares psychological aspects commonly related to non-substance addictions, such as problematic gaming and social media behavior. When intoxication through a substance, such as drugs or alcohol, is taken out of the addiction algorithm, the dependency paradox becomes even more puzzling, but perhaps more interesting psychology-wise. As shown repeatedly in, for example, smoking cessation research (Killen et al., 1992), the psychological dependency is considerably stronger than the physiological one. Thus, the fact that exercise dependence was differently related to the two escapism mindsets in the present study may have relevance to the understanding of the psychological *drive* in other types of addictive behaviors, both substance and non-substance addictions. Somewhat surprising, also Self-expansion was positively correlated to exercise dependence, which shows that addiction-like engagement in running may embed nourishing psychological experiences. This is, at a second glance, perhaps not surprising after all. Even those addicted to an activity find pleasure in the engagement in the activity, to the sense that it becomes impossible to abstain from it, and the positive aspects of escapism may be one such addiction-promoting psychological motive.

The present study supports the notion that activity engagements may be motivated from different motivational mindsets. The two-dimensional mode of escapism applies regulatory focus theory (Higgins, 1998) to illuminate promotion and prevention mindsets in relation to a particular activity, and further how these sets associate with different cognitive and affective experiences, and consequences, in the activity engagement, as well as to general well-being. As such, the escapism model departs from what normally is the primary focus in coping research, which most often assesses coping as a temperamental trait without reference to a particular activity. There are, however, several overlaps between, for example, Roth and Cohens’ model of coping (1986), which also is of a dualistic nature, with approach coping and avoidance coping as antagonistic components. Of relevance, Stenseng et al. (2021)study 2 showed that participants’ tendencies for avoidance coping were primarily related to Self-Suppression when using streaming services, whereas approach coping was only related to Self-Expansion. Thus, there appears to be an overlap between trait emotion coping and escapism. Future studies should look more closely into how coping mechanisms are related to the escapism mindsets embedded in the two-dimensional model of escapism.

Escapism is, by many authors as well as in bibliographical definitions, described as a shift away from everyday life. Thus, the term does not only entail the state of escape in an activity, such as in the case of flow, but is interpreted as a more holistic perspective of everyday functioning. Escapism (divergence) is seen as a contrast to what is outside (routine); thus, a causal interplay between these two “worlds,” so to speak, may emerge. A cascade model may apply, in which lower well-being leads to a stronger self-suppression mindset in the activity, which undermines positive effect and the potentially nourishing experiences in the activity, which then inflicts negatively on life outcomes. This relates to findings in the present study, where Self-Suppression was more strongly related to both exercise dependence and general subjective ill-being, compared to Self-Expansion. Exercise dependence undermines the potential positive psychological consequences from running, but notably, lower general subjective well-being may be seen as both a cause and an outcome of such dependency. One short-term (repeated measures) study found that an increase in general negative effect predicted increase in self-suppression over time (Stenseng et al., 2012, Study 3), but much remains to be investigated regarding how escapism and activity engagements evolve over time in a day-to-day scenario.

## Limitations

The present study has several limitations. First, the convenience sample, recruited though several digital channels, may not be representative of recreational runners in general. Nevertheless, derived from the descriptive sample statistics, one may conclude that very few participants were competitive runners, and the gender balance in the sample was rather even. Second, only one measure of escapism was used, and given that escapism in running is a rather uninvestigated theme, a multi-scale approach to the phenomenon would have added more strength to the ecological validity of the findings. Third, subdimensions of exercise dependence were not analyzed in the present study. Although this was beyond the scope of the present study objectives, a deeper look into which subdimensions of exercise dependence overlap most and least with the escapism dimensions might be fruitful. Fourth, the cross-sectional design of the present study makes it impossible to draw any inferences regarding the causal interplay of escapism, exercise dependence, and well-being. Hopefully, this will be addressed more thoroughly in future studies using longitudinal research designs.

## Conclusion

Although prominent researchers such as Baumeister (1991) have highlighted escapism as a fundamental aspect of human motivation and emphasized the dualism to it, a sound operationalization of these stipulations has largely failed to emerge. The two-dimensional model of escapism, with its corresponding scale, may be a pathway toward more empirical scrutiny of this term. In the present study, related to recreational running, the validity of the scale was supported, and its two subdimensions had meaningful correlations to exercise dependence and subjective well-being. In sum, the present findings suggest escapism is a relevant phenomenon in exercise, covering both adaptive and maladaptive aspects of escapism motivation.

## Data availability statement

The raw data supporting the conclusions of this article will be made available by the authors, without undue reservation.

## Ethics statement

Ethical approval was not provided for this study on human participants because this study contained no personal identifiable information. The patients/participants provided their written informed consent to participate in this study.

## Author contributions

All authors listed have made a substantial, direct, and intellectual contribution to the work and approved it for publication.

## Conflict of interest

The authors declare that the research was conducted in the absence of any commercial or financial relationships that could be construed as a potential conflict of interest.

## Publisher’s note

All claims expressed in this article are solely those of the authors and do not necessarily represent those of their affiliated organizations, or those of the publisher, the editors and the reviewers. Any product that may be evaluated in this article, or claim that may be made by its manufacturer, is not guaranteed or endorsed by the publisher.
